# Nomadic Tibetan women’s reproductive health: findings from cross-sectional surveys with a hard-to-reach population

**DOI:** 10.1186/s12978-020-01052-0

**Published:** 2021-03-17

**Authors:** Jessica D. Gipson, Corrina Moucheraud, Kunchok Gyaltsen, Lumo Tsering, Tabashir Z. Nobari, Lhusham Gyal

**Affiliations:** 1 Fielding School of Public Health, University of California, 650 Charles E. Young Drive South, Los Angeles, CA 90095 USA; 2 Tso-Ngon (Qinghai) University Tibetan Medical College, No. 251 Ningda Road, Xining, 810000 Qinghai People’s Republic of China; 3 Department of Public Health, California State University, 800 North State College, Boulevard, KHS 131, Fullerton, USA

**Keywords:** China, Tibetan, Nomad, Reproductive health

## Abstract

**Background:**

Western China has undergone substantial sociodemographic change, yet little is known about the health status of ethnic minority populations living in these areas.

**Methods:**

We report findings from two cross-sectional surveys conducted with female Tibetan nomads living in rural areas of Western China/Eastern Tibet. We present results of descriptive analyses of data collected from reproductive-aged females who attended community health fairs in 2014 (n = 193) and 2016 (n = 298).

**Results:**

On average, sexual debut preceded marriage among study participants, with fertility near replacement levels (2.7 and 2.1 in 2014 and 2016, respectively). Contraceptive use was common, and dominated by use of IUDs and female sterilization. Although over three-quarters (76%) of 2016 survey participants reported ever having at least one sexually transmitted infection (STI) symptom, there was low awareness of STIs (59%) and action to prevent STIs (21%). Younger women (< 40) were more likely to report having had had an STI symptom, as compared to older women (84% versus 71%; p < 0.05).

**Conclusions:**

We demonstrate feasibility of collecting data with this hard-to-reach population. Reporting of STI symptoms warrants further investigation to identify and address health conditions in this population of Tibetan nomadic women, especially amidst broader social and contextual changes that may affect the Tibetan population.

## Plain English Summary

Western China has undergone substantial sociodemographic change, yet little is known about the health status of ethnic minority populations living in these areas. We report findings from two cross-sectional surveys conducted with female Tibetan nomads living in rural areas of Western China/Eastern Tibet. We present results of descriptive analyses of data collected from reproductive-aged females who attended community health fairs in 2014 (n = 193) and 2016 (n = 298). On average, sexual debut preceded marriage among study participants, with fertility near replacement levels (2.7 and 2.1 in 2014 and 2016, respectively). Contraceptive use was common, and dominated by use of IUDs and female sterilization. Although over three-quarters (76%) of 2016 survey participants reported ever having at least one sexually transmitted infection (STI) symptom, there was low awareness of STIs (59%) and action to prevent STIs (21%). Younger women (less than 40 years old) were more likely to report having had had an STI symptom, as compared to women over 40 years old (84% versus 71%; p < 0.05). We demonstrate feasibility of collecting data with this hard-to-reach population. Reporting of STI symptoms warrants further investigation to identify and address health conditions in this population of Tibetan nomadic women, especially amidst broader social and contextual changes that may affect the Tibetan population.

## Introduction

Western China has historically been home to a large number of ethnic minority residents, but has undergone substantial economic and social change in the past two decades [1, 2]. Government initiatives have prompted economic development in Western China, including the expansion of private industry, leading to an influx of Han (ethnic majority) Chinese into the more rural areas of China and the Tibetan plateau [1, 2]. In addition to these development efforts, policies that promote the settlement of Tibetan populations into urban areas have substantially altered the cultural context of Western China. Despite these myriad changes, these provinces remain some of the poorest in China [3]. Little is known about how ethnic minority populations have fared amidst these sociodemographic shifts [4].

Previous analyses document persistent disparities in health outcomes across Chinese provinces (e.g., maternal mortality; child health) [5, 6], yet there have been fewer investigations of ethnic minority populations in these more-disadvantaged areas [7]. Very little is known about nomadic Tibetan populations, likely due to their combined identity as an ethnic minority group, and as a mobile, difficult-to-reach population. While some evidence has shown health advantages among nomadic populations (e.g., biological adaptations to higher altitudes) [8, 9], other studies indicate that rural ethnic minority populations are more vulnerable to health issues given their distance from health facilities, extreme climate and terrain, linguistic and cultural barriers, and challenging lifestyle and environmental factors (e.g., availability of food and water) [4, 10, 11]. Ongoing political and ethnic tensions may also impede health care-seeking behavior and affect the quality of care provided for Tibetan nomads. Ethnic minority populations may face unique barriers, including communication with health professionals at Chinese health care facilities and discriminatory treatment [12]. Evidence suggests that these nomadic groups may even experience differential treatment by other Tibetans [13]. Despite the challenges, however, a recent analysis of maternal health behaviors among Tibetans in Qinghai Province found that utilization of antenatal care, institutional delivery, and/or skilled birth attendance has significantly increased over time, from 10% of births prior to the year 2000, to approximately 50% between 2000 and 2014 [14]. Moreover, women were more likely to use these services if they participated in the New Cooperative Medical Scheme, the national health insurance plan available to Chinese citizens [15].

Among the few studies that have examined the sexual and reproductive health status of Tibetan populations [5, 16–19], a focus on nomadic populations is rare. Although Tibetan and other minority populations in China were granted some leniency to China’s One-Child Policy (implemented by the Chinese Government in the late 1970s and early 1980s to limit population growth by promoting one child per family), the 2000 census indicated below-replacement fertility levels in the Tibetan autonomous prefectures and somewhat higher in the two most remote nomadic prefectures (Qinghai tabulation 2002: Table L.6–1 by Fischer, 2008). In-depth examinations of fertility and family planning practices in Tibetan communities underscore the sensitivity of these topics, given historical evidence of coercive practices and challenges in reconciling official family planning policy with Tibetan beliefs and practices [1, 19]. However, ethnographic studies by Schrempf [13] and Craig [20] have explored these topics—and describe the sociocultural significance of motherhood and fertility for Tibetan women, including the importance of continuing the family lineage, maintaining karma, and ensuring a woman’s status in her partnership and her community [13]. Additionally, contraceptive methods and their documented side effects may run counter to Tibetan cultural schemas surrounding the importance of hard work and strength among Tibetan women, and the extent to which women are knowledgeable about how contraceptives work in the body especially given low education levels, awareness of methods, and discrepancies between Western and Tibetan health beliefs [13].

Examinations of sexual health—including sexually transmitted infections—are extremely limited in ethnic minority populations, and particularly among nomadic populations. National estimates suggest that the prevalence of sexually transmitted infections (STIs) has increased in recent decades [21], and some studies have found relatively higher risks for ethnic minority populations versus Han groups [22–24].

This project sought to document the reproductive health status of an ethnic minority population—Tibetan nomads—living in Qinghai province in Western China. Qinghai Province is part of the traditional Tibetan region of Amdo and has the third largest Tibetan population outside of the Tibetan Autonomous Region and Sichuan (Fischer 2008). Qinghai is consistently ranked below the China average in per capita income and GDP [3]. In 2014 and 2016, a team of public health and medical practitioners collected data from Tibetan nomads living in two areas of Western China to assess their reproductive health status. A separate paper by Moucheraud et al. [14] examined maternal health care behaviors using the 2014 data. Following on those findings, the aim of this paper is to integrate the 2014 and 2016 data to report on the reproductive health status and behaviors of Tibetan nomadic women across these two data collection efforts in this remote, ethnic minority population.

## Materials and methods

**Fig. 1:**
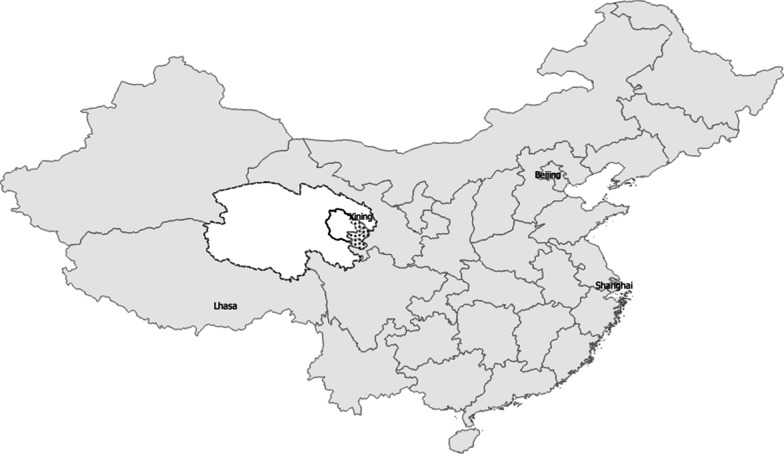
Map of study areas in Qinghai Province

The two surveys—Site A (2014) and Site B (2016)—were conducted in rural areas of Western China/Eastern Tibet by faculty and students from the Tso-Ngon (Qinghai) University Tibetan Medical College (TUTMC) in Tso-ngon, Tibet/Qinghai, China and (See map; Fig. 1).

Due to the difficulty of collecting data from this hard-to-reach, rural and nomadic population, data were collected during 2–3 day community health fairs in two different Tibetan prefectures: Site A in 2014 and Site B in 2016 [14]. (We subsequently refer to these sites as Site A and Site B). The health fairs were advertised via the County Health Department and Women’s Union, as well as by Tibetan village leaders and village doctors in the area. The health fairs were open to anyone who attended and drew approximately 1600 people per fair.

During these health fairs, male and female participants were offered free medical consultation from Tibetan and Chinese health professionals and health education sessions; however, only adult women were asked to participate in the health survey. In total, 200 women at Site A and 306 women at Site B consented to participate in the survey.

All data collectors for the two surveys were Tibetan medical students or professionals who were trained on the administration of the survey, including pretesting the surveys with Tibetan students and refining the survey instruments prior to implementation in the field. The two surveys were developed by a team of Tibetan-US researchers to include questions about respondents’ birth histories, health care seeking, health beliefs, and general sociodemographic characteristics, including selected questions from the Demographic Health Survey modules [25]. The surveys were conducted face-to-face in the local language (Amdo Tibetan).

The final analytic samples were Site A: n = 193 (7 women reported not ever having had sex) and Site B: n = 298 (2 women were missing on age; 6 women reported not ever having had sex). All analyses were conducted using Stata version 15. Bivariate associations were assessed using t-tests and Chi-squared tests; a p value of 0.05 or less was considered statistically significant. A sum score representing ownership of four items (motorcycle, car, radio, and television) served as a proxy for household wealth. Questions regarding sexually transmitted infections (STIs) were introduced into the Site B survey following input from Tibetan colleagues and health care providers involved with the Site A health fair.

### Ethical consideration

Ethical clearance for data collection was provided by TUTMC. Participants were interviewed individually in a separate area from the health fair. The survey was explained to each woman and, due to limited literacy in this population, were asked to provide their oral consent. Following the surveys, the data were maintained by the lead investigator at TUTMC in password-protected files and fully anonymized. The de-identified information was provided to investigators at University of California, Los Angeles (UCLA) for secondary data analysis. The UCLA Office of the Human Research Protection Program determined that analysis of these data did not meet the definition of human subjects research, so exempt this study from review.

## Results

Table 1 displays the sociodemographic and reproductive characteristics of the two samples. The samples predominantly consist of women in older reproductive ages (Site A mean = 36.9 years; Site B mean = 41.0 years). At both sites, most women reported being currently married, and approximately 5% reported never being married and 10% reported being divorced or widowed. The vast majority of women reported no formal education (80% and 72% in Site A and Site B, respectively); of those women who reported any education, women reported 4–5 years on average. Households reported modest levels of household wealth and the vast majority were enrolled in the New Cooperative Medical Scheme, [15].

**Table 1:** Sociodemographic characteristics of Tibetan Nomadic Women, 2014 and 2016

	2014 Site A	2016 Site B
	n = 193	N = 298
Sociodemographic characteristics		
Mean age of respondents (range; median)	36.9 (21–50; 37) years	41.0 (18–55; 42) years
Marital status		
Never married	5.2%	4.4%
Married	82.4%	86.2%
Divorced	8.8%	5.1%
Widowed	3.6%	4.4%
Educational attainment		
% reporting 0 years	80.2%	72.0%
Average number of years (median)^a^	5.5 years (6)	4.3 years (4)
Household wealth (asset score: 0–4)	1.9 (0–4; 0.8)	2.2 (0–4; 0.7)
Enrollment in New Cooperative Medical System (NCMS), %	83.3%	83.9%
Pregnancy and birth histories	Mean (range; median)	
Average number of pregnancies	3.0 (1–7; 3)	2.4 (0–8; 2)
Average number of live births	2.7 (0–6; 3)	2.1 (0–6; 2)
Age at first sex	17.1 (11–24; 17)	18.2 (7–27; 18)
Age at first birth	21.0 (16–33; 20)	20.8 (13–37; 20)
Ever had a miscarriage, %	16.1%	10%
Ever had an abortion^b^	0.5%	14.5%
Contraceptive use		
Report ever use of contraception	94.3%	83.5%
Report current use of contraception	82.9%^c^	55.9%^d^
IUD	73.8%	77.4%^e^
Tubal ligation^f^	22.5%	8.8%
Pill	1.3%	6.6%
Condom	1.9%	5.8%
Diaphragm	0.6%	–
Injection	–	1.5%

^a^Among women with any education

^b^Questions were asked differently between the two surveys. The 2014 survey asked women to report “How many times a pregnancy ended with an abortion?” The 2016 survey asked women to report each pregnancy first, then to report the outcome of each pregnancy

^c^Survey did not ascertain current pregnancy status of women

^d^N = 250 who reported current contraceptive use. Omitted 5 women who were currently pregnant (n = 245)

^e^2 women who reported IUD and condom use were included in the IUD category

^f^Two women who reported current use of injection and tubal ligation were recoded as having had a tubal ligation

Women reported 2–3 lifetime pregnancies and births, on average, with women from the Site A sample reporting more pregnancies and births on average, as compared to the Site B sample (3.0 vs. 2.4 for pregnancies; 2.7 vs. 2.1 for live births, respectively). Ages at first sex and first birth were similar across the two sites, although women in Site A reported a larger delay between first sex and first birth (17.1 and 21.0 years, average delay of 3.2 years) as compared to women in Site B (18.2 and 20.8 years, average delay of 2.6 years). Differences in the questions regarding miscarriage and abortion across the two surveys prevent comparison across sites; however, 14.5% of women in the Site B survey and 0.5% of women in the Site A survey reported ever having an abortion.

Women reported high levels of ever using contraception (94% and 84% in Site A and Site B, respectively), and of current use (83% and 56%, respectively). Both populations indicate high current reliance on intrauterine devices (IUDs) and tubal ligation, as compared to other contraceptive methods. Reported use of the pill, condom, and injection were low, especially in Site A.

Data on awareness of STIs and possible STI-related symptoms were collected in the Site B survey only (Table 2). Just over half (58.6%) of the respondents reported they were aware that some infections and diseases may be transmitted sexually. In total, 76% of women reported ever experiencing any STI symptom, most commonly vaginal discharge (59.2%), itchiness (50.6%), and painful urination (20.6%). Twenty-one percent of women reported ever doing anything to prevent an STI.

**Table 2:** Reported awareness of STIs and STI symptoms among Tibetan nomadic women, Site B County 2016

	Total	Women < 40 (n = 118)	Women 40+ (n = 180)
Awareness of STIs	59%	55%	61%
Ever experienced any STI symptom	76%	84%	71%*
Ever experienced			
Painful urination	21%	23%	19%
Itchiness	51%	52%	50%
Discharge	59%	75%	47%***
Painful sex	11%	14%	7%^†^
Genital warts	17%	19%	17%
Ever done anything to prevent STI	21%	26%	18%
Number of lifetime sexual partners	2.0 (1–15; 1.0)	2.2 (1–10; 2.0)	1.9 (1–15; 1.0)^†^
Number of sexual partners in last month	0.9 (0–2; 1)	1.0 (0–2; 1)	0.9 (0–1; 1)***

t-test of difference: ^†^p < 0.1, *p < 0.05, **p < 0.01, ***p < 0.001

We examined whether there were reported differences in the STI measures in the Site B survey between older women (40 years and above) and younger women (less than 40 years). Compared to older women, younger women were significantly more likely to report ever experiencing an STI symptom (p < 0.05) (Table 2). When looking at specific symptoms, experience of discharge and painful sex were more likely among younger versus older women.

Further examination of a potential risk factor—number of sexual partners—revealed that there was a marginally significant difference in the average number of lifetime sexual partners reported by women 40 years and above (2.2) versus women less than 40 years (1.9); however, significantly more women 40 years and above had only 1 lifetime partner, as compared to younger women (less than 40 years) (57.0% versus 43.1%, respectively, p value < 0.05). The average number of recent sexual partners (within the last month) was significantly less among women 40 years and above (0.9 partners) than women less than 40 years old (1 partner) (p value < 0.001).

## Discussion

This paper provides descriptive sociodemographic and reproductive health data collected from a hard-to-reach population—Tibetan nomads in Western China. Due to the sparse distribution of the population and their remote location, we implemented a novel and unique way of gathering data from women via community health fairs. This enabled both men and women from this underserved population to access health services, while also providing a venue for collecting basic health information.

Examination of the sociodemographic characteristics of these two samples indicate low education levels among participating women (72–80% reported no formal education). These levels are lower than those of women who participated in a separate study in a nearby agricultural area [26, 27], yet similar to educational levels documented in another study of nomadic Tibetan women [4]. Women’s education is a critical component in improving women’s status within societies and in achieving optimal health outcomes for women and their families [28, 29]. Improved opportunities for schooling, including for women and girls, are critical to achieving better health outcomes and well-being, while also maintaining Tibetan cultural identity and language [30].

Fertility levels reported among these samples (2.7 and 2.1 live births) likely approximate total fertility rates given that a large proportion of women from these samples were likely to have completed childbearing. (Additional analyses of Site B data did not indicate significant cohort differences in fertility between women less than 40 years versus women 40 years and above) Of note is that in both populations, sexual debut was reported to be at least two years prior to reported age at marriage and first birth, indicating that premarital sex is occurring and perhaps with the use of contraception. This finding is in contrast to some other Asian populations and aligns with past ethnographic research on the “loose association” between marriage and fertility in Tibetan societies ([31–33]—from Spoorenberg) and may suggest that young unmarried women are able to access contraception to delay pregnancy. It is also important to consider, however, the extent to which subfecundity or infertility may affect the ability to conceive (at any stage of the reproductive lifespan) given historical evidence of higher rates of primary and secondary infertility among Tibetan versus Han populations [34, 35].

The surveys did not collect data on types of contraception used throughout the lifecourse; however, women in the sample who reported current use of contraception predominantly relied on IUDs and tubal ligations. China has a history of high-reliance on IUDs and female sterilization [36]. While these methods are highly effective in preventing pregnancy and may be the preferred methods for this lower-fertility, remote population, it is also important that women (and men) have access to the full range of desired methods and high-quality contraceptive services [37]. We were not able to ascertain from the data the extent to which other contraceptive methods are used by younger women who wish to delay or space their pregnancies using short-term or reversible contraceptive methods.

Over three-quarters (76%) of the 2016 survey sample indicated that they had experienced at least one symptom that can be associated with STIs. It is important to note that while these symptoms are included in international surveys as a means of possible identification of STI infection, they are not highly specific to STIs and may reflect other infections or morbidities (e.g., urinary tract infection) [38]. It is also important to interpret these self-reported results cautiously—especially given the potential stigma and ongoing challenges with the validity of self-reported measures of sexual behavior across global settings [39].

Despite these measurement challenges; however, the significantly higher reporting of “ever” having an STI-related symptom among younger versus older women is notable which may reflect differential risk, as suggested by the marginally significant and higher mean number of lifetime sexual partners among younger women (2.2) as compared to older women (1.9). Alternately, it is possible that there may be different levels of comfort and reporting of number of sexual partners between younger and older women.

Although the community health fair provided an innovative approach to data collection among this marginalized population, there are challenges to this approach. Namely, we did not know how many women would attend the community health fair, nor did we know the characteristics of the women who would be able to or would be most willing to attend. The samples were older than expected, with mean ages of 37 (Site A) and 41 (Site B) at the two sites. Although the age of the sample allowed us to collect data on a range of reproductive experiences and history, in this relatively low-fertility population, for a proportion of the women in the sample, these reproductive experiences occurred nearly two decades prior.

The findings from this exploratory study should also be considered within the context in which the data were collected—although the health fair provided a venue for data collection among this marginalized population, data collected at the health fair selectively reflect the characteristics of women who chose to (and were able to) attend the health fair and who agreed to participate in the survey. Given the difficulty of reaching this marginalized population, this study provides initial insights on the health status and areas for continued investigation. Moreover, if we consider that that women who attended these health fairs may have had greater resources or capacity to attend such an event, it is possible that sociodemographic and health indicators (e.g., low levels of educational attainment and STI awareness) could be even lower within the broader population.

Further studies are needed to determine if the substantial environmental and structural changes in Western China may be altering risk exposure to STIs among ethnic minority populations. Rises in STI and HIV incidence have been documented in other settings marked by economic growth and expansion of transportation [40], and migration [41]. In addition to these broader contextual changes, there are aspects of the Tibetan lifestyle that are also changing—anecdotal data indicate that Tibetans in this region are increasingly employed in construction and road work projects that require them to move frequently, especially as other economic options (e.g., harvesting caterpillar fungus) become less accessible. In addition, our data collection effort was unfortunately limited to women and, thus, we are unable to comment on the potential change in risk and sexual behavior among Tibetan nomadic men.

## Conclusions

Overall, findings from this study demonstrate the feasibility of collecting data via health fairs among this hard-to-reach, remote, ethnic minority population. While timing of reproductive events was relatively stable across the age cohorts, high reporting of STI symptoms warrants further investigation to identify and address health conditions in this population of Tibetan nomadic women, especially amidst broader social and contextual changes that may affect the risk profiles of the Tibetan population.

## Data Availability

The datasets generated during and/or analysed during the current study are not publicly available due to the confidential and sensitive nature of the study population; however, these data may be made available from the corresponding author on reasonable request.

## Abbreviations

STI: Sexually transmitted infection
IUDs: Intrauterine devices
